# Impurity Location-Dependent Relaxation Dynamics of Cu:CdS Quantum Dots

**DOI:** 10.1186/s11671-017-1832-3

**Published:** 2017-01-18

**Authors:** Dayeon Choi, Ji-Young Pyo, Du-Jeon Jang

**Affiliations:** Department of Chemistry, Seoul National University, NS60, Seoul, 08826 Republic of Korea

**Keywords:** Doping, Energy transfer, Impurity position, Quantum dots, Relaxation dynamics

## Abstract

Various types of 2% Cu-incorporated CdS (Cu:CdS) quantum dots (QDs) with very similar sizes have been prepared via a water soluble colloidal method. The locations of Cu impurities in CdS host nanocrystals have been controlled by adopting three different synthetic ways of doping, exchange, and adsorption to understand the impurity location-dependent relaxation dynamics of charge carriers. The oxidation state of incorporated Cu impurities has been found to be +1 and the band-gap energy of Cu:CdS QDs decreases as Cu_2_S forms at the surfaces of CdS QDs. Broad and red-shifted emission with a large Stokes shift has been observed for Cu:CdS QDs as newly produced Cu-related defects become luminescent centers. The energetically favored hole trapping of thiol molecules, as well as the local environment, inhibits the radiative recombination processes of Cu:CdS QDs, thus resulting in low photoluminescence. Upon excitation, an electron is promoted to the conduction band, leaving a hole on the valence band. The hole is transferred to the Cu^+^ d-state, changing Cu^+^ into Cu^2+^, which then participates in radiative recombination with an electron. Electrons in the conduction band are ensnared into shallow-trap sites within 52 ns. The electrons can be further captured on the time scale of 260 ns into deep-trap sites, where electrons recombine with holes in 820 ns. Our in-depth analysis of carrier relaxation has shown that the possibilities of both nonradiative recombination and energy transfer to Cu impurities become high when Cu ions are located at the surface of CdS QDs.

**Electronic supplementary material:**

The online version of this article (doi:10.1186/s11671-017-1832-3) contains supplementary material, which is available to authorized users.

## Background

With the great growth of interest in the field of nanochemistry, the study of nanocrystalline semiconductors has become increasingly important over a few decades due to their unique optical and electrical properties compared with the respective ones of bulk semiconductors [1, 2]. Transition-metal or rare-earth ion-doped quantum dots (QDs), especially sulfides, are emerging as alternatives to semiconductor QDs with stable, strong, and tunable luminescence in the visible spectral region for different optoelectronic applications. Minimized self-absorption [3, 4], long excited-state lifetimes [5, 6], tunable emission spectral widths [7], and thermal stability [8, 9] are the characteristic properties of these nanocrystals making those doped nanocrystals as important QDs. Especially, semiconductor nanocrystals of a wide band-gap such as cadmium or zinc chalcogenides, doped with transition-metal ions, become alternative materials to overcome the limitation of organic phosphor-based LEDs (OLEDs) because QD-LEDs do not suffer from the spin statistics that limits the internal quantum efficiency of fluorescent OLEDs [10]. The incorporation of transition-metal dopants like Mn, Cu, and Co ions introduces intermediate energy states, such as trap states, between the valence and the conduction bands of host semiconductor nanocrystals and influences the relaxation dynamics of host materials [9, 11–14]. As a consequence, the dopant emission results in unique optical properties whose nature varies with chosen hosts and dopants. Copper-doped nanocrystals have been reported to have long-lived excited states with the suppressed intrinsic band-edge emission of host nanocrystals [7].

It has been a challenge to find out the optimized synthetic route of doping, providing extremely small volumes of nanocrystals only even with vigorous organometallic approaches. Therefore, it is necessary to find new strategies to incorporate dopant ions to the host nanocrystals [15]. However, the synthesis of highly luminescent and structurally stable QDs in a large quantity via a reproducible way still remains as a challenge. Peng et al. has designed two different strategies: nucleation doping and growth doping [4]. In the nucleation-doping strategy, the formation of the host nanocrystals has been reported to occur under established synthetic conditions and then to be quenched by lowering the reaction temperature [4]. Under the new conditions, active dopant precursors were introduced and doping took place without the growth of the host. Growth doping was realized by mixing dopant and host precursors during nucleation. After nucleation, the reaction conditions were tuned to be sufficiently mild to make the dopant precursor inactive, and the growth of the host became the only process, over-coating the dopant. Although the doping strategy has affected the dopant locations and the host optical properties, little research has been achieved to reveal the influence of dopant locations on the luminescence properties of host QDs systematically.

**Fig. 1:**
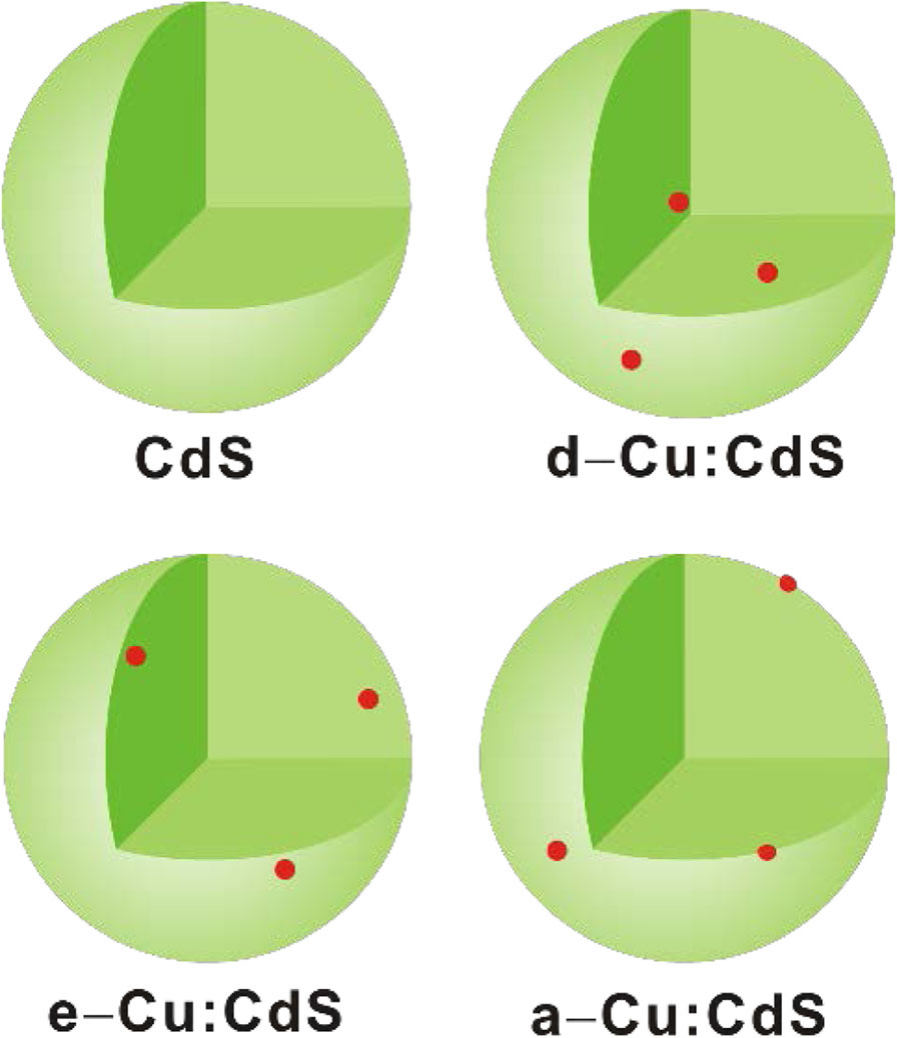
Schematic representation of pristine (CdS), 2% Cu-doped (d-Cu:CdS), 2% Cu-exchanged (e-Cu:CdS), and 2% Cu-adsorbed CdS (a-Cu:CdS) QDs, where the *red dots* represent Cu impurities

Here, we have prepared three types of Cu:CdS QDs having Cu ions at different locations by controlling the synthetic ways of doping, exchange, and adsorption (Fig. 1) to understand the impurity location-dependent relaxation dynamics of charge carriers in the prepared QDs. We have studied their emission properties by correlating photoluminescence quantum yields (PLQYs) with the efficiency of energy transfer from an exciton inside the CdS host to a Cu ion (*Φ*_ET_) and the efficiency of emission from the Cu ion (*Φ*_Cu_). Also, we have obtained photoluminescence (PL) decay curves by using time-resolved spectroscopy and extracted PL decay time constants by fitting the decay curves with multi-exponential decay functions. Finally, we have tried to correlate surface effects and dopant positions in QDs with the optical properties of Cu:CdS QDs.

## Methods

### Preparation

#### Cd^2+^-2-Mercaptopropionic Acid (MPA) and Cu^2+^-MPA Stock Solutions

A Cd^2+^/MPA stock solution, where the molar ratio of Cd^2+^/MPA was 1/2.5, was prepared by adding 6.25 mmol of MPA into 100 mL of 0.025 M Cd(NO_3_)_2_(aq) while a Cu ^+^/MPA stock solution, where the molar ratio of Cu^2+^/MPA was 1/2.5, was prepared by adding 1.25 mmol of MPA into 20 mL of 0.025 M CuCl_2_(aq). Each stock solution was adjusted to pH 7.5 using 2.0 M NaOH(aq), diluted five times with water, and then stored in the dark at room temperature until needed.

#### CdS QDs

Fifty milliliter of the Cd^2+^-MPA stock solution was placed into a four-necked round-bottom flask and bubbled with N_2_(g) for 30 min under vigorous stirring to remove O_2_(g). After the solution was heated to 100 °C, it was quickly added with 1.0 mL of 0.125 M Na_2_S(aq) and allowed to react for 4 h at 100 °C. Note that the molar ratio of Cu^2+^/S^2−^/MPA in the final reaction mixture was 1/0.5/2.5. The product was precipitated with 100 mL of 2-propanol, centrifuged at 10,000 rpm for 30 min, and redispersed in 50 mL of water.

#### d-Cu:CdS QDs

The molar ratio of Cd^2+^/Cu^2+^/S^2−^/MPA in the final reaction mixture of Cu:CdS QDs was 0.98/0.02/0.5/2.5. In order to synthesize d-Cu:CdS QDs, 1.0 mL of the Cu^2+^-MPA stock solution was added to 49 mL of the Cd^2+^-MPA stock solution and then the solution was bubbled with N_2_(g) for 30 min under vigorous stirring. When the mixture was heated to 100 °C, it was added with 1.0 mL of 0.125 M Na_2_S(aq) quickly and allowed to react for 4 h at 100 °C. The actual composition of d-Cu:CdS QDs measured via inductively coupled plasma atomic emission spectroscopy (ICP-AES) has been found to be Cd_0.984_Cu_0.016_S_1.090_.

#### e-Cu:CdS QDs

Forty-nine milliliter of the Cd^2+^-MPA stock solution was placed into a flask and bubbled with N_2_(g) for 30 min under vigorous stirring. After the solution was heated to 100 °C, it was added with 1.0 mL of 0.125 M Na_2_S(aq) quickly and allowed to react for 2 h at 100 °C. The prepared colloidal solution of CdS QDs was added with 1.0 mL of the Cu^2+^-MPA stock solution and allowed for cation exchange to take place at 100 °C for additional 2 h.

#### a-Cu:CdS QDs

1.0 mL of the Cu^2+^-MPA stock solution and 1.0 mL of 0.125 M Na_2_S(aq) were added to 49 mL of the prepared colloidal solution of CdS QDs and allowed to react for 2 h under vigorous stirring and N_2_(g) bubbling at 100 °C.

### Characterization

PLQYs were measured by comparing the integrated PL intensities of QDs with those of primary standard rhodamine B (PLQY = 65% in ethanol) solutions excited at the same wavelength of 355 nm. The integrated intensities of the emission spectra, corrected for differences in refraction indices, were used to calculate PLQYs. High-resolution transmission electron microscopy (HRTEM) images were obtained using a JEOL JEM-3000F microscope, and X-ray diffraction (XRD) patterns were obtained with a Bruker D8 DISCOVER diffractometer. Absorption spectra were monitored with a Scinco S-3100 spectrophotometer. Detailed procedures for the measurement of emission spectra and PL decay profiles have already been reported [1]. The composition of d-Cu:CdS QDs was detected with a Perkin-Elmer OPTIMA 4300DV ICP-AES spectrometer. The valence state of the Cu element in the Cu:CdS QDs was characterized using a Bruker EMX-plus X-band CW electron paramagnetic resonance (EPR) spectrometer.

## Results and Discussion

**Fig. 2:**
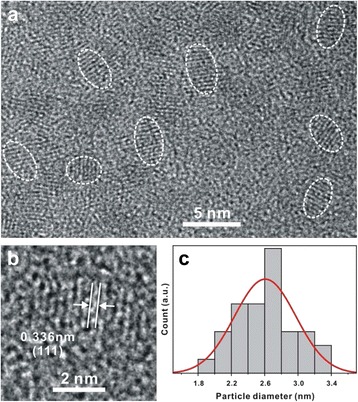
**a** HRTEM image, **b** enlarged HRTEM image, and **c** particle-size histogram of e-Cu:CdS QDs

Figure 2 shows the HRTEM images of e-Cu:CdS QDs prepared by exchanging the 2% Cd^2+^ ions of CdS QDs with Cu^2+^ ions. The cation exchange reaction is considered to take place spontaneously because the radii of Cd^2+^ and Cu^2+^ ions are 95 and 73 pm, respectively, and the *K*_sp_ values of CdS, CuS, and Cu_2_S at room temperature are 7 × 10^−28^, 8 × 10^−37^, and 2 × 10^−47^, respectively. From the *K*_sp_ values, the calculated standard Gibbs free energies of the expected exchange reactions CdS(s) + Cu^2+^(aq)→CuS(s) + Cd^2+^(aq) and CdS(s) + 2Cu^2+^(aq)→Cu_2_S(s) + Cd^2+^(aq) at 100 °C are −64 and −2900 kJ mol^−1^, respectively. The HRTEM image of Fig. 2a indicates that as-prepared e-Cu:CdS QDs have elliptical structures. The observed lattice-fringe distance in Fig. 2b is 0.336 nm, which agrees well with the standard spacing of 0.336 nm between the adjacent (111) lattice planes of the cubic zinc-blende crystal of CdS. Figure 2c and Table 1 indicate that the average value of the major and the minor axes calculated from HRTEM images of the e-Cu:CdS QDs is 2.6 ± 0.4 nm. The diameters of the elliptical QDs have been calculated by averaging the major and the minor axes of the QDs. The HRTEM images of Fig. 2 and Additional file 1: Figure S1 indicate that all the different types of our prepared Cu:CdS QDs have very similar elliptical structures and sizes as given in Table 1. The lattice-fringe distances observed from the enlarged HRTEM images of Fig. 2 and Additional file 1: Figure S1 also suggest that the structural details of host CdS QDs have hardly been modified by the incorporation of 2% Cu ions.

**Table 1:** Calculated particle sizes, deconvoluted emission spectra, and PLQYs of various QDs dispersed in water

Sample	Size^a^/nm	Size^b^/nm	Size^c^/nm	*d*_spacing_^d^/nm	*E*_g_^e^/eV	*λ*_em_^f^/nm	*λ*_1_	*λ*_2_	*λ*_3_	QY/%
CdS	2.3	2.8	3.2	0.336	3.01	501	465 ± 28(12%)^g^	504 ± 47(40%)	569 ± 68(48%)	9.1
d-Cu:CdS	2.5	2.6	3.4	0.364	2.91	632	499 ± 54(14%)	631 ± 67(76%)	749 ± 56(10%)	5.3
e-Cu:CdS	2.6	2.8	3.5	0.362	2.89	635	506 ± 67(17%)	634 ± 67(71%)	740 ± 63(12%)	3.4
a-Cu:CdS	2.6	2.7	3.8	0.354	2.79	646	647 ± 79(93%)	788 ± 57(7%)	0.5	

^a^Average value of the major and the minor axes calculated from HRTEM images

^b^Average diameter calculated from an XRD pattern

^c^Diameter calculated from an absorption spectrum using the Brus equation

^d^Average distance between adjacent (111) planes, observed from an XRD pattern

^e^Band-gap energy in the unit of nm

^f^Wavelength at the emission maximum

^g^Area percentage

**Fig. 3:**
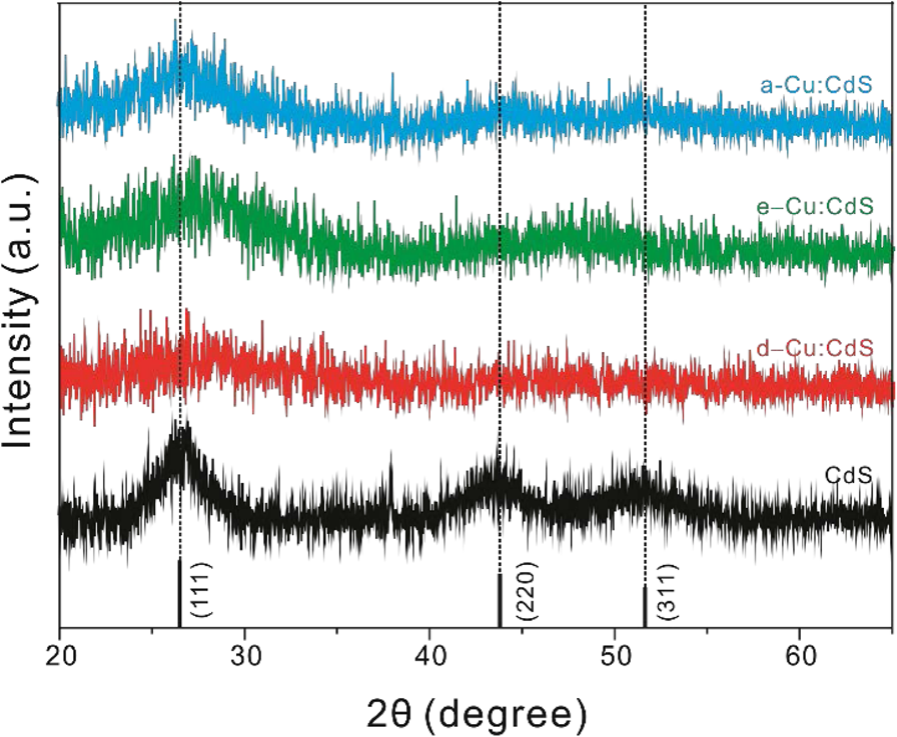
XRD patterns of Cu:CdS QDs, whose types are indicated inside. The reference XRD pattern of CdS is also shown at the bottom for comparison

The XRD patterns in Fig. 3 also indicate that the crystals of Cu:CdS QDs have the cubic zinc-blende structure, where the standard XRD pattern (JCPDS No. 00-010-0454) is shown at the bottom. The 2*θ* values of the (111), (220), and (311) planes coming from the CdS host crystal have hardly been affected by the presence of Cu impurities. However, in the case of d-Cu:CdS, the peaks are shapeless because the existence of Cu ions at the initial stage of the crystal growth has affected the crystallinity due to lattice strains induced by difference in the lattice constants of CdS (5.81 Å) from those of CuS (3.80 Å) or Cu_2_S (5.76 Å). As a result, the (111) peaks of the three incorporated samples are dramatically wider than that of the pure sample. The mean crystallite sizes, calculated from XRD line-broadening widths at 2*θ* of 26.5° using the Scherrer’s equation [3], have been estimated as 2.6~2.8 nm, which match well with the values calculated from the HRTEM data (Table 1). Average distances between the adjacent (111) planes of Cu:CdS QDs, obtained with the Bragg’s law, have been calculated to be 0.336~0.364 nm (Table 1). Thus, our results indicate that the lattice structures of Cu:CdS QDs are almost the same regardless of the locations of Cu impurities.

**Fig. 4:**
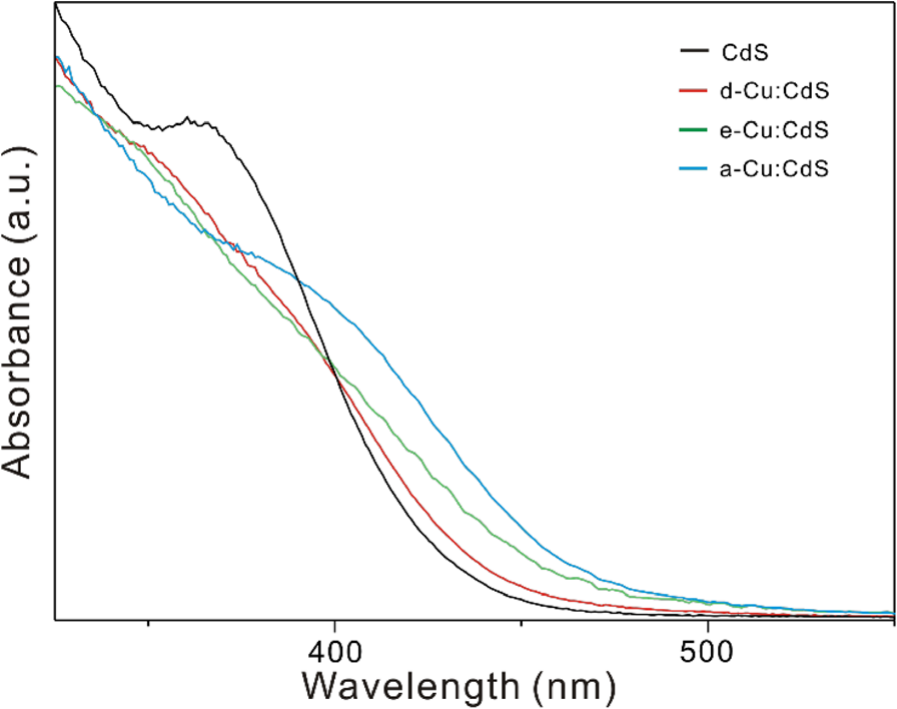
Absorption spectra of indicated QDs dispersed in water

Given that the band-gap of the bulk CdS is 2.49 eV (498 nm) and that the Bohr exciton radius of CdS is 3.0 nm, the absorption onset of CdS QDs at 370 nm (3.35 eV) in Fig. 4 is clearly blue-shifted and indicative of the quantum confinement effect. The excitonic peak at 370 nm has not been shifted by the incorporation of 2% Cu ions, although there is a slight red-shift in a-Cu:CdS QDs due to the formation of CuS (2.2 eV) or Cu_2_S (1.2 eV) at the surface of CdS QDs. Therefore, it has been demonstrated that the 2% Cu incorporation hardly affects the absorption spectrum of host CdS QDs because photon absorption arises mainly from the CdS host. We have obtained the band-gaps (*E*_g_) of Cu:CdS QDs by using the modified Kubelka-Munk plots from the absorption spectra of Fig. 4. The relationship between the absorption coefficients (*α*) near the absorption edge and *E*_g_ for direct interband transitions is known to obey (*α*hν)^2^ = *A*(hν − *E*_g_), where *A* is a parameter and hν is the photon energy [1]. By extrapolating the linear portions of the (*α*hν)^2^ plots versus hν to 0 in Additional file 1: Figure S2, we have found that the *E*_g_ of CdS, d-Cu:CdS, e-Cu:CdS, and a-Cu:CdS QDs are 3.01, 2.97, 2.89, and 2.79 eV, respectively (Table 1). This suggests that *E*_g_ decreases gradually as CuS or Cu_2_S having a smaller *E*_g_ forms at the surface of CdS QDs [14].

**Fig. 5:**
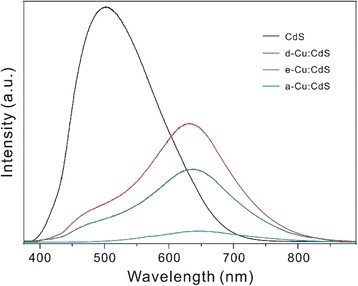
Emission spectra of indicated QDs dispersed in water. The samples were excited at 355 nm

Figure 5 shows that for CdS QDs, defect-related emission with a large Stokes shift (~130 nm) is dominant while band-edge emission is negligibly weak. Thus, the emission, as well as absorption, spectra are typical of CdS QDs prepared by a colloidal method without surface modification [11]. The energy difference between the maxima of absorption (370 nm, 3.35 eV) and emission spectra (501 nm, 2.48 eV) has been mainly attributed to trap sites within the band-gap [16]. It is known that many nanocrystal systems can have significant concentrations of Schottky and Frenkel defects. Also, the impurities could be a part of luminescent centers by forming complexes with host crystals. These newly made defects may also be trap states that lead to emission. Furthermore, the broader emission band of Cu:CdS QDs has been attributed to the transition of electrons from the conduction band of the host to the energy level of Cu impurity. Especially, with MPA as a capping agent, the hole-trapping effect of the thiol group has been reported; the hole trapping of a thiol on a CdS QD is energetically favorable because the redox energy level of a thiol is situated at a higher energy than the valence-band top of CdS [17]. In the case of Cu:CdS QDs, PL emission is drastically red-shifted because band-edge emission is highly suppressed by Cu-related emission and by the hole-trapping effect [14, 18–20]. Also, the PLQY of Cu:CdS QDs is drastically reduced due to the hole-trapping effect; the hole is trapped on a thiol molecule, resulting in a severely reduced PLQY (Table 1). Overall, it can be deduced that PL emission is shifted largely (by 145 nm) to a longer wavelength and PLQY decreases drastically (by a factor of 18) as 2% Cu ions are incorporated at the surface of CdS QDs.

In order to understand the energy levels of as-prepared QDs, each emission spectrum has been deconvoluted with Gaussian functions, as shown in Additional file 1: Figure S3. In the case of CdS QDs, the raw emission peak is asymmetric and fitted with three Gaussian curves of *λ*_1_ at 465 ± 28 nm, *λ*_2_ at 504 ± 47 nm, and *λ*_3_ at 569 ± 68 nm (Table 1). The *λ*_1_ peak is assigned to the near band-edge emission; the band gap of CdS QDs is 3.01 eV (411 nm) and the observed Stokes shift of our as-prepared CdS QDs is about 50 nm, which is a typical value of the Stokes shift observed in CdS QDs prepared by a colloidal method without surface modification [11]. Electrons in the conduction band descend to shallow-trap states or impurity donors and then recombine with holes in the valence band or hole trap states, resulting in the PL of the *λ*_2_ peak around 504 nm. Finally, the *λ*_3_ peak arises from deep-trap states which stem from cadmium vacancies or sulfur dangling bonds on the surface, resulting in emission around 569 nm. Compared with the respective ones of CdS QDs, whereas the *λ*_1_ peak of d-Cu:CdS (e-Cu:CdS) QDs is somewhat red-shifted by 34 (41) nm, the *λ*_2_ and *λ*_3_ peaks of d-Cu:CdS (e-Cu:CdS) QDs are highly red-shifted by 127 (130) and 180 (171) nm, respectively. This suggests that whereas the *λ*_1_ peak arises from host CdS QDs, both *λ*_2_ and *λ*_3_ peaks are attributed to emission from trap states to dopant states. For a-Cu:CdS QDs, as the band-edge emission of CdS QDs has been quenched completely, PL has been deconvoluted with only two Gaussian functions of *λ*_2_ and *λ*_3_. This suggests that CuS or Cu_2_S formed at the surface of CdS QDs, in particular, quenches the host emission efficiently (see below).

The identification of the oxidation states of copper ions in the CdS crystals is very important to understand the carrier relaxation process. As Cu^+^ (d^10^) and Cu^2+^ (d^9^) have different electronic configurations, electron paramagnetic resonance (EPR) spectra have also been measured. No EPR signals have been observed in our as-prepared Cu:CdS QDs, suggesting that Cu^2+^ ions are not present at all (Additional file 1: Figure S4) [21]. However, the doping precursor used in our work is Cu^2+^, whose valence is the same as the valence of Cd^2+^ ions in CdS QDs. Thus, we presume that the valence of a copper ion has been changed from 2+ to 1+ during the incorporation process, as the reduction of Cu^2+^ to Cu^+^ by anions (sulfide ions) present in CdS QDs has already been proposed [21]. Note that the lattice mismatch (0.83%) of Cu_2_S (lattice constant, 5.762 Å) with CdS (lattice constant, 5.810 Å) is much smaller than that (35%) of CuS (lattice constant, 3.802 Å). This suggests that the formation of Cu_2_S is energetically much more favorable than that of CuS in the lattice of CdS QDs.

**Fig. 6:**
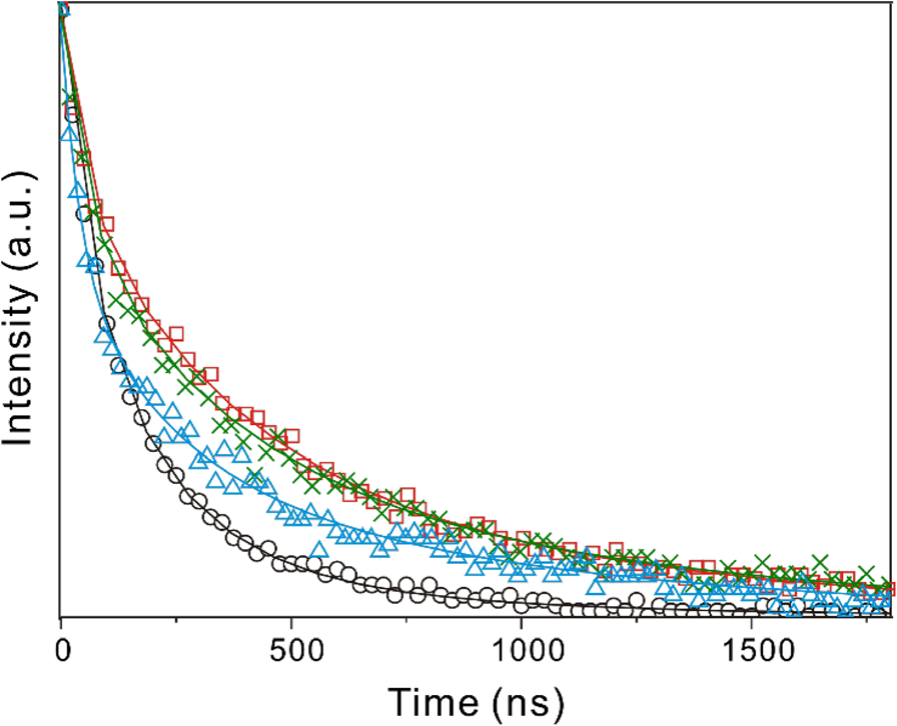
PL decay curves of CdS (*circles*), d-Cu:CdS (*squares*), e-Cu:CdS (*crosses*), and a-Cu:CdS (*triangles*) QDs dispersed in water. The samples were excited at 355 nm and monitored at 650 nm. The *solid lines* are best-fitted curves

Figure 6 and Table 2 show that the PL decay curves of the prepared QDs collected at 650 nm can be fitted tri-exponentially. While the fast decay component of 52 ns is attributed to the band-edge emission of the *λ*_1_ peak, the medium component is ascribed to the shallow-trap emission of the *λ*_2_ peak and the slow component to the deep-trap emission of the *λ*_3_ peak. The mean lifetimes of Cu:CdS QDs are much larger than that of pristine CdS QDs. In particular, the lifetimes of both the medium and the slow components have been increased extensively by the incorporation of 2% Cu ions, suggesting that electrons in shallow- and deep-trap sites combine slowly with holes in copper states to yield the largely red-shifted and long-lived trap emission of the *λ*_2_ and *λ*_3_ peaks. Thus, the internal crystals structure and the charge-carrier relaxation dynamics of CdS QDs are suggested to depend highly on the locations of Cu ions due to lattice strains. Therefore, d-Cu:CdS QDs, where Cu ions have been introduced at the initial stage of the crystal growth, have the longest PL decay time than any other types of Cu:CdS QDs.

**Table 2:** PL decay time constants at 650 nm of various QDs dispersed in water

Sample	*τ*/ns	<*τ*>/ns
CdS	52(56%) + 110(24%) + 315(20%)^a^	118
d-Cu:CdS	52(25%) + 294(35%) + 824(40%)	446
e-Cu:CdS	52(18%) + 272(49%) + 824(33%)	415
a-Cu:CdS	52(28%) + 210(48%) + 824(24%)	313

^a^Initial amplitude percentage of each component

**Fig. 7:**
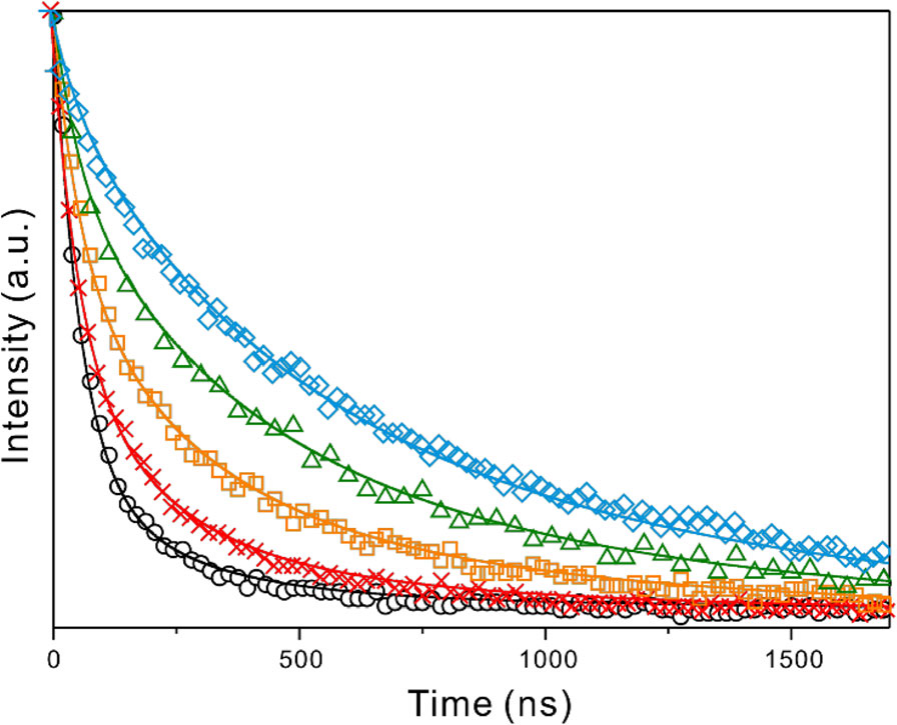
PL decay curves of d-Cu:CdS QDs. The samples were excited at 355 nm and monitored at (*circles*) 500, (*crosses*) 550, (*squares*) 600, (*triangles*) 650, and (*diamonds*) 700 nm. The solid lines are best-fitted curves

We have studied the relaxation dynamics of Cu:CdS QDs systematically by monitoring the wavelength-dependent emission decay profiles of d-Cu:CdS QDs (Fig. 7). Table 3 shows that as the PL wavelength increases, the mean PL lifetime becomes longer because the relative amplitude percentage of the slow component becomes larger; whereas the largest component is the fast component at 500 nm, it becomes the medium one and the slow one at 600 and 700 nm, respectively. Considering Figs. 6 and 7 together with Fig. 5 and Additional file 1: Figure S3, we can conclude that for d-Cu:CdS QDs suspended in water, the band-edge emission of the CdS host at 499 nm decays within 52 ns, emission from a shallow-trap of CdS to a Cu impurity level at 631 nm decays in 294 ns, and emission at 749 nm from a deep-trap of CdS to a Cu impurity level decays on the time scale of 824 ns [19].

**Table 3:** Wavelength-dependent PL decay time constants of d-Cu:CdS QDs dispersed in water

Sample	*λ*_mon_/nm	*τ*/ns	<*τ*>/ns
d-Cu:CdS	500	52(80%) + 294(18%) + 824(4%)	111
	550	52(58%) + 294(39%) + 824(3%)	170
	600	52(36%) + 294(49%) + 824(15%)	286
	650	52(25%) + 294(35%) + 824(40%)	446
	700	52(11%) + 294(27%) + 824(62%)	596

The PL properties of Cu-doped nanoparticles have been extensively studied [18–21]; it is well known that the dopant luminescence of these nanoparticles is owing to energy transfer from excitons to Cu states [22]. Following Cao et el. [23], we have expressed the QY of the band-gap PL (QY_BG_), the efficiency of energy transfer to Cu (*Φ*_ET_), the efficiency of emission from a Cu ion (*Φ*_Cu_), and the QY of the Cu PL (QY_Cu_). The different types of Cu-doped QDs are suggested to have an almost identical density of surface-trap states, and these QDs have a nearly identical core size and the same Cu-doping level because they have been prepared using a similar thermal colloidal method at the same precursor concentration. Therefore, we can assume reasonably that the nonradiative-relaxation rate constants (*k*_BG-NR_) are identical in different Cu:CdS QDs. We can also suppose that the radiative-relaxation rate constants (*k*_BG-R_) are identical in our Cu:CdS QDs. Based on these two assumptions, we can obtain Eq. (1) and (2).

1 $$ {\varPhi}_{\mathrm{ET}} = 1-\frac{{\mathrm{QY}}_{\mathrm{BG}}}{{\mathrm{QY}}_{\mathrm{UD}}} $$

2 $$ {\varPhi}_{\mathrm{Cu}} = {\mathrm{QY}}_{\mathrm{Cu}}/{\varPhi}_{\mathrm{ET}} $$

Thus, *Φ*_ET_ and *Φ*_Cu_ have been calculated using the QY_BG_ and QY_Cu_ of the doped QDs as well as the QY_UD_ of undoped CdS QDs, as shown in Table 4.

**Table 4:** Exciton PLQY (QY_ex_), Cu PLQY (QY_cu_), energy-transfer efficiency (*Φ*_ET_), and Cu PL efficiency (*Φ*_Cu_) of various QDs dispersed in water

Sample	QY/%	QY_ex_/%	*Φ*_ET_/%	*Φ*_Cu_/%	QY_Cu_/%
CdS	9.1	9.1			
d-Cu:CdS	5.3	0.9	90.9	4.84	4.4
e-Cu:CdS	3.4	0.6	93.5	2.99	2.8
a-Cu:CdS	0.5	0.0	100	0.5	0.5

Considering the sizes of Cu:CdS QDs (2.3~2.6 nm), the doping concentration of Cu impurities (2% only), and the lattice constant of CdS (5.81 Å) together, we can suggest that Cu ions have been incorporated in the host CdS nanocrystals in an isolated manner regardless of impurity locations. Among our various types of Cu:CdS QDs, a-Cu:CdS QDs have the lowest QY and *Φ*_Cu_ values whereas they have the highest *Φ*_ET_ value. The symmetry and the local strain of the local coordinating environment of a Cu ion mainly determine the radial-position-dependent behavior of *Φ*_Cu_ [23]. The surface effects of nanocrystals lead to an increase in the asymmetry of the local coordinating environment of a Cu impurity, decreasing *Φ*_Cu_ drastically. And, as mentioned above, it is another reason that the hole-trapping effect of a thiol on a QD is energetically favored. Upon excitation, electrons in the conduction band go down to the bottom of the conduction band promptly, and then they are ensnared in trap sites. Coincidently, holes in the valence band go up to the redox level of a thiol located right above the valence-band top of CdS. Therefore, the probability of radiative recombination is low and the possibilities of both nonradiative recombination and energy transfer to Cu impurities are high when Cu ions are located at the surface of CdS QDs. The QY_Cu_ of Cu:CdS QDs can also be strongly influenced by the hole-trapping effect. In nanocrystals, it has been widely accepted that the outer part of nanocrystals is more easily exposed to the environment such as solvent molecules than the inner part of them is. Therefore, we have demonstrated that Cu dopants in a-Cu:CdS QDs are mostly located in the surfaces of CdS host nanocrystals, compared with those in the other types of Cu:CdS QDs. The cation exchange, which extensively occurs from the surface of CdS QDs, makes the dopants located near the surface in e-Cu:CdS QDs [24]. Although d-Cu:CdS QDs have the lowest *Φ*_ET_ value, they show the highest QY_Cu_ value as Cu ions are isolated at the relatively inner part of the CdS host. It is interesting to note that although a-Cu:CdS QDs have the highest *Φ*_ET_ value, they have the lowest QY_cu_ due to the hole-trapping effect. We have concluded that the presence of Cu impurities at relatively different locations quenches CdS host emission via energy transfer to Cu impurities and that energetically favored hole trapping in a-Cu:CdS QDs inhibits the overall radiative recombination processes very effectively.

## Conclusions

**Fig. 8:**
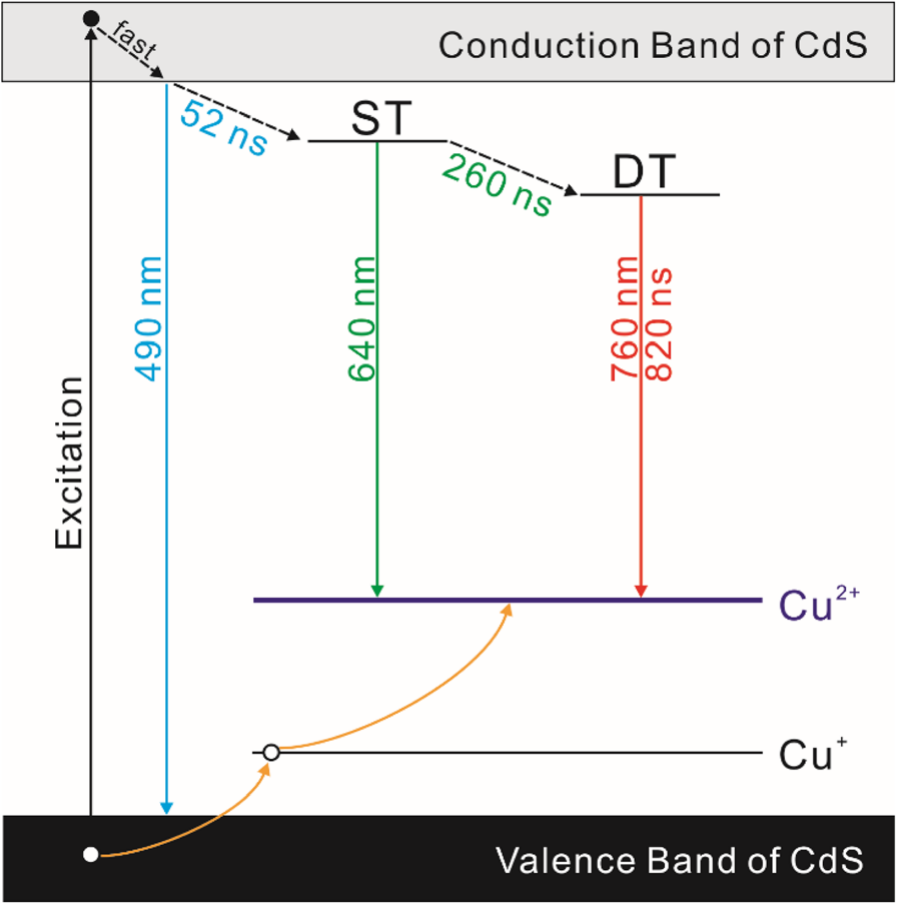
Schematic mechanism of the impurity location-dependent carrier relaxation dynamics of Cu:CdS QDs

In summary, three different types of 2% Cu-incorporated CdS QDs having nearly similar sizes have been prepared via a water soluble colloidal method. The locations of Cu impurities in CdS host nanocrystals have been controlled by adopting three different synthetic ways of doping, exchange, and adsorption to understand the impurity location-dependent relaxation dynamics of charge carriers. The crystallinity of CdS host nanocrystals has been affected by incorporated Cu ions due to lattice mismatches, the oxidation state of incorporated Cu impurities has been found to be +1, and the band-gap energy of Cu:CdS QDs gradually decreases as Cu_2_S forms at the surfaces of CdS QDs. The broader emission band with a large Stokes shift has been observed for Cu:CdS QDs as newly produced Cu-related defects play an important role in the emission process. The energetically favored hole trapping of thiol molecules on the QDs inhibits the overall radiative recombination processes of Cu:CdS QDs when Cu ions are isolated at the surfaces of CdS QDs, thus resulting in low PLQYs [25]. Taking all the presented results into consideration, we propose the mechanism of the impurity location-dependent carrier relaxation in Cu:CdS QDs (Fig. 8). Being distinct from optically active Cu^2+^ (d^9^), the d-states of Cu^+^ have been filled with electrons (d^10^). Therefore, Cu^+^ is optically passive and cannot contribute to emission unless it captures a hole from the valence band of the CdS host. Upon excitation, an electron located at the valence band of CdS QDs is promoted to the conduction band, leaving a hole on the valence band. As the d-state of Cu^+^ and the valence band of the CdS QDs are located energetically nearby, the hole could be transferred to the Cu^+^ d-state, changing Cu^+^ into Cu^2+^, which then participates in radiative recombination with an electron [26, 27]. Once electrons are excited to the conduction band, the electrons descend swiftly to the bottom of the conduction band of CdS, emitting band-edge PL at 490 nm until they are ensnared into shallow-trap (ST) sites within 52 ns. The electrons in ST sites can be further captured into deep-trap (DT) sites on the time scale of 260 ns. Electrons trapped into ST and DT sites recombine with holes in the dopant states at 640 and 760 nm, respectively. The relaxation time of electrons in DT sites has been found as 820 ns. Our in-depth analysis of carrier relaxation has shown that probability of radiative recombination is low and that the possibilities of both nonradiative recombination and energy transfer to Cu impurities are high when Cu ions are located at the surface of CdS QDs.

## Additional file

Additional file 1: Figure S1. HRTEM images (left) and enlarged HRTEM images (right) of (a) CdS, (b) d-Cu:CdS, and (c) a-Cu:CdS QDs. **Figure S2.** Modified Kubelka-Munk plots of (a) CdS, (b) d-Cu:CdS, (c) e-Cu:CdS, and (d) a-Cu:CdS, where the calculated band-gap of each d-dot is indicated inside. **Figure S3.** Emission spectra (black) of (a) CdS, (b) d-Cu:CdS, (c) e-Cu:CdS, and (d) a-Cu:CdS QDs dispersed in water, fitted with three Gaussian curves of *λ*_1_ (blue), *λ*_2_ (green), and *λ*_3_ (red). **Figure S4.** EPR spectra of d-Cu:CdS, e-Cu:CdS, and a-Cu:CdS QDs.

## Abbreviations

BG: Band-gap
DT: Deep trap
EPR: Electron paramagnetic resonance
ET: Energy transfer
HRTEM: High-resolution transmission electron microscopy
MPA: Mercaptopropionic acid
NR: Nonradiative
OLEDs: Organic phosphor-based light emitting diode
PL: Photoluminescence
PLQYs: Photoluminescence quantum yields
QD: Quantum dots
R: Radiative
ST: Shallow trap
XRD: X-ray diffraction
