# Supplementary material for: Impurity Location-Dependent Relaxation Dynamics of Cu:CdS Quantum Dots
Source: Nanoscale Res Lett. 2017 Jan 18;12:49. doi: 10.1186/s11671-017-1832-3 (PMC5241571; doi:10.1186/s11671-017-1832-3)
Supplement: Additional file 1: Figure S1. — HRTEM images (left) and enlarged HRTEM images (right) of (a) CdS, (b) d-Cu:CdS, and (c) a-Cu:CdS QDs. Figure S2. Modified Kubelka-Munk plots of (a) CdS, (b) d-Cu:CdS, (c) e-Cu:CdS, and (d) a-Cu:CdS, where the calculated band-gap of each d-dot is indicated inside. Figure S3. Emission spectra (black) of (a) CdS, (b) d-Cu:CdS, (c) e-Cu:CdS, and (d) a-Cu:CdS QDs dispersed in water, fitted with three Gaussian curves of λ 1 (blue), λ 2 (green), and λ 3 (red). Figure S4. EPR spectra of d-Cu:CdS, e-Cu:CdS, and a-Cu:CdS QDs. [file 11671_2017_1832_MOESM1_ESM.docx]

**Supplementary Materials**

Impurity Location-Dependent Relaxation Dynamics of

Cu:CdS Quantum Dots

Dayeon Choi, Ji-Young Pyo, and Du-Jeon Jang*

*Department of Chemistry, Seoul National University, NS60, Seoul 08826, Korea*

E-mail: [djjang@snu.ac.kr](mailto:djjang@snu.ac.kr)


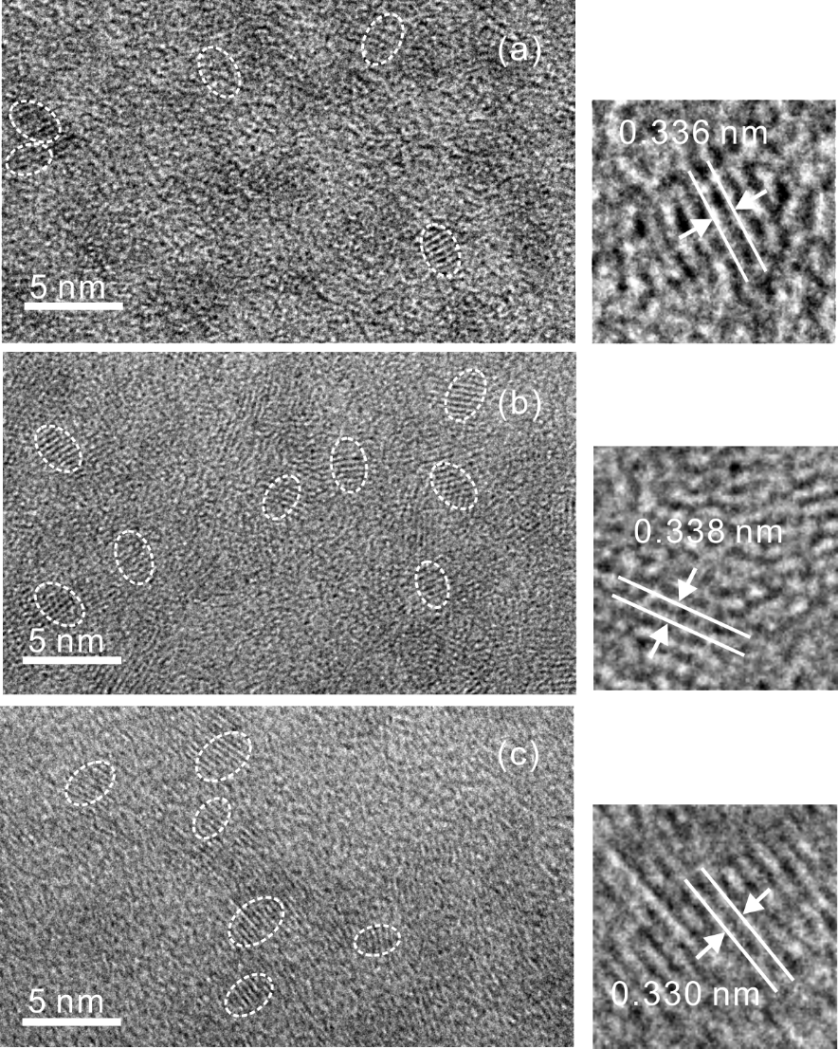


**Figure S1** HRTEM images (left) and enlarged HRTEM images (right) of (a) CdS, (b) d-Cu:CdS, and (c) a−Cu:CdS QDs.


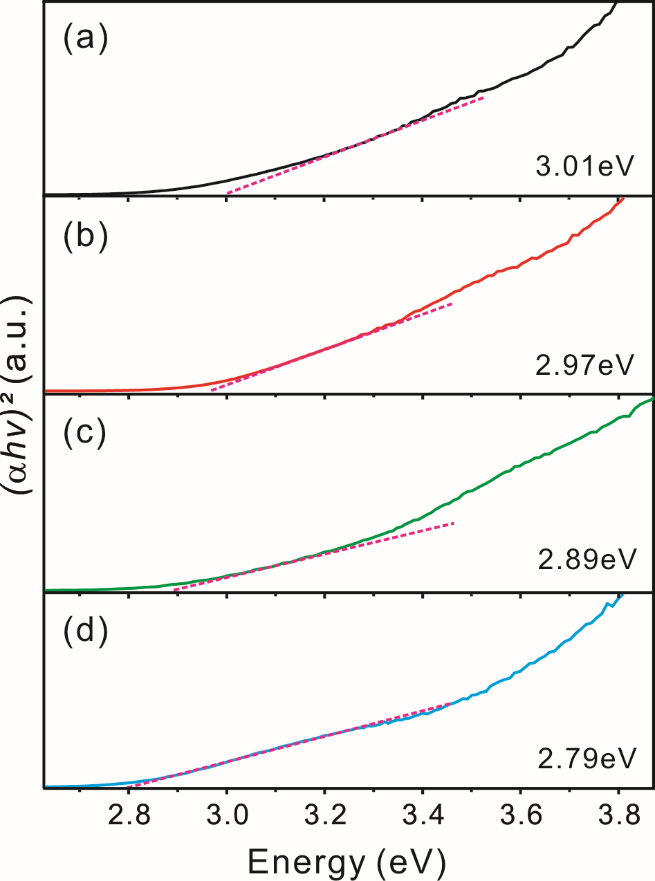


**Figure S2** Modified Kubelka-Munk plots of (a) CdS, (b) d−Cu:CdS, (c) e−Cu:CdS, and (d) a−Cu:CdS, where the calculated band gap of each d-dot is indicated inside.


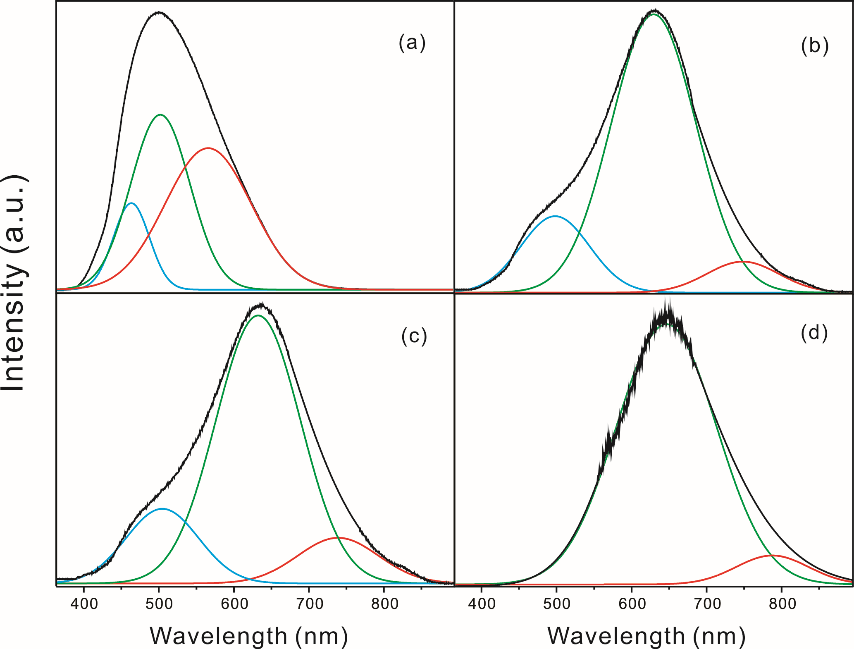


**Figure S3** Emission spectra (black) of (a) CdS, (b) d-Cu:CdS, (c) e-Cu:CdS, and (d) a-Cu:CdS QDs dispersed in water, fitted with three Gaussian curves of λ_1_ (blue), λ_2_ (green), and λ_3_ (red).


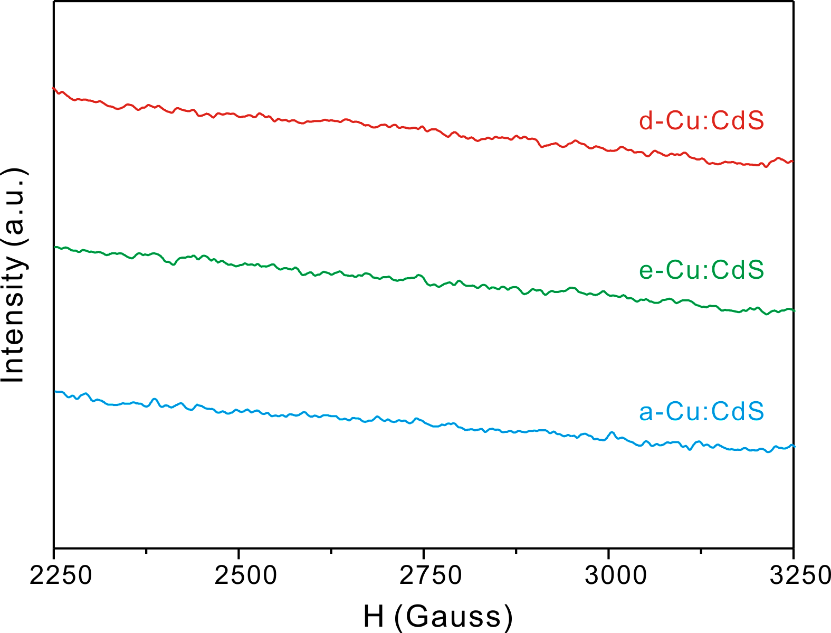


**Figure S4** EPR spectra of d-Cu:CdS, e-Cu:CdS, and a-Cu:CdS QDs.
